# Artesunate treatment ameliorates ultraviolet irradiation-driven skin photoaging via increasing β-catenin expression

**DOI:** 10.18632/aging.203749

**Published:** 2021-12-09

**Authors:** Liming Tian, Dan Ke, Yi Hong, Chong Zhang, Daizhi Tian, Long Chen, Lirui Zhan, Shiqin Zong

**Affiliations:** 1 Department of Dermatology, Wuhan No.1 Hospital, Hospital of Traditional Chinese and Western Medicine Affiliated to Hubei University of Chinese Medicine, Wuhan Hospital of Traditional Chinese and Western Medicine Affiliated to Huazhong University of Science and Technology, Wuhan 430022, Hubei, China; 2 Department of Dermatology, Chongqing Traditional Chinese Medicine Hospital, Chongqing 400000, China; 3 College of Pharmacy, Hubei University of Chinese Medicine, Wuhan 430065, Hubei, China; 4 Institute of Geriatrics, Hubei University of Chinese Medicine, Wuhan 430065, Hubei, China

**Keywords:** artesunate, skin photoaging, β-catenin, ultraviolet radiation, cell senescence

## Abstract

Objective: Artesunate, a semi-synthetic derivative of artemisinin, exerts various pharmacological activities. Nevertheless, the effects of Art on skin photoaging remain unclear. Herein, we investigated whether Art ameliorated ultraviolet-irradiated skin photoaging in HaCaT cells and mice.

Methods: To construct skin photoaging cellular models, HaCaT cells were irradiated by UV (UVB, 20mJ/cm^2^) for 5 days. HaCaT cells were pretreated with three concentrations of Art (1, 5 and 20 μg/ml) for 2 h each day. After 5 days, cell senescence, ROS production, SOD levels, p16^INK4a^ and β-catenin expression, proliferation and apoptosis were detected in HaCaT cells. Effects of Art on normal cells were investigated. After sh-β-catenin transfection or XAV-939 treatment, HaCaT cells were pretreated with 20 μg/ml Art and irradiated by UVB. After 5 days, skin photoaging was then observed. Furthermore, skin photoaging mouse models were established and the effects of Art and β-catenin silencing on skin photoaging were investigated.

Results: Art treatment suppressed cell senescence, intracellular ROS production, p16^INK4a^ expression and apoptosis and promoted proliferation and SOD and β-catenin expression in UVB irradiated HaCaT cells. But Art had no toxic effects on normal cells. Silencing β-catenin by sh-β-catenin or XAV-939 exacerbated UVB irradiation-mediated cell senescence, apoptosis, and ROS production in HaCaT cells, which was ameliorated by Art treatment. The therapeutic effects of Art on skin photoaging were also confirmed in mouse models.

Conclusions: These findings suggested that Art treatment alleviated UVB irradiation-driven skin photoaging through enhancing β-catenin expression, which offered novel clues for pharmacological activity of Art.

## INTRODUCTION

Skin photoaging represents a badly complex and coordinated biological event [1–3]. Once photoaging begins, collagen fibers will degrade, resulting in sagging skin and wrinkles [4]. In addition, abnormal proliferation of melanocytes may contribute to skin pigmentation [5–7]. Long-term ultraviolet (UV) radiation that is emitted naturally from the sun and artificial sources is the main cause of photoaging [8]. UVB (λ=280-320nm) is a common ultraviolet ray that causes skin aging, and studies have shown that 20-144 mJ/cm^2^ UBV can cause cell photoaging [9–11], and 55-200 mJ/cm^2^ UBV can induce mouse skin aging [10, 12, 13]. Increasing evidence suggests that UV radiation initiates skin photoaging through inducing oxidative stress and reactive oxygen species (ROS) production, thereby destroying cell macromolecules like protein, lipid, and DNA [14–16]. Enzymatic antioxidant superoxide dismutase (SOD) is an essential component of the skin defense against ROS-induced injury [17, 18]. However, the excessive production of ROS caused by UV exposure may weaken the skin's endogenous antioxidant ability and induce collagen and elastin fiber degradation, ultimately leading to skin aging [19, 20]. Decreasing the accumulation of intracellular ROS has become an effective strategy for preventing UV-mediated cell senescence and skin photoaging.

Artesunate (Art) is a semi-synthetic derivative of artemisinin [21]. It has attracted more and more attention due to its anti-malarial, anti-oxidant, anti-inflammatory, anti-cancer and other biological activities and pharmacological safety [22]. Studies have reported that Art may suppress tumor progression (such as osteosarcoma [23], uveal melanoma [24] and colorectal carcinoma [25]), liver fibrosis [26] and myelodysplastic syndromes [27] via blocking β-catenin pathway. Moreover, limited evidences suggest that Art has the potential to treat skin-related diseases. For instance, Art ameliorates 2, 4-dinitrochlorobenzene-induced atopic dermatitis through down-regulation of Th17 cell response [28]. Additionally, it can also relieve imiquimod-induced psoriasis-like dermatitis [29]. However, the therapeutic effects of Art skin photoaging remain unclear. Herein, this study investigated that Art exerted an inhibitory effect on ultraviolet-irradiated skin photoaging in HaCaT cells and mouse models. β-catenin was responsible for the therapeutic effects of Art. Collectively, our study provided novel clues for pharmacological activity of Art.

## RESULTS

### Art treatment reduces cell senescence, intracellular ROS production and increases SOD expression in UV-irradiated HaCaT cells

**Figure 1:**
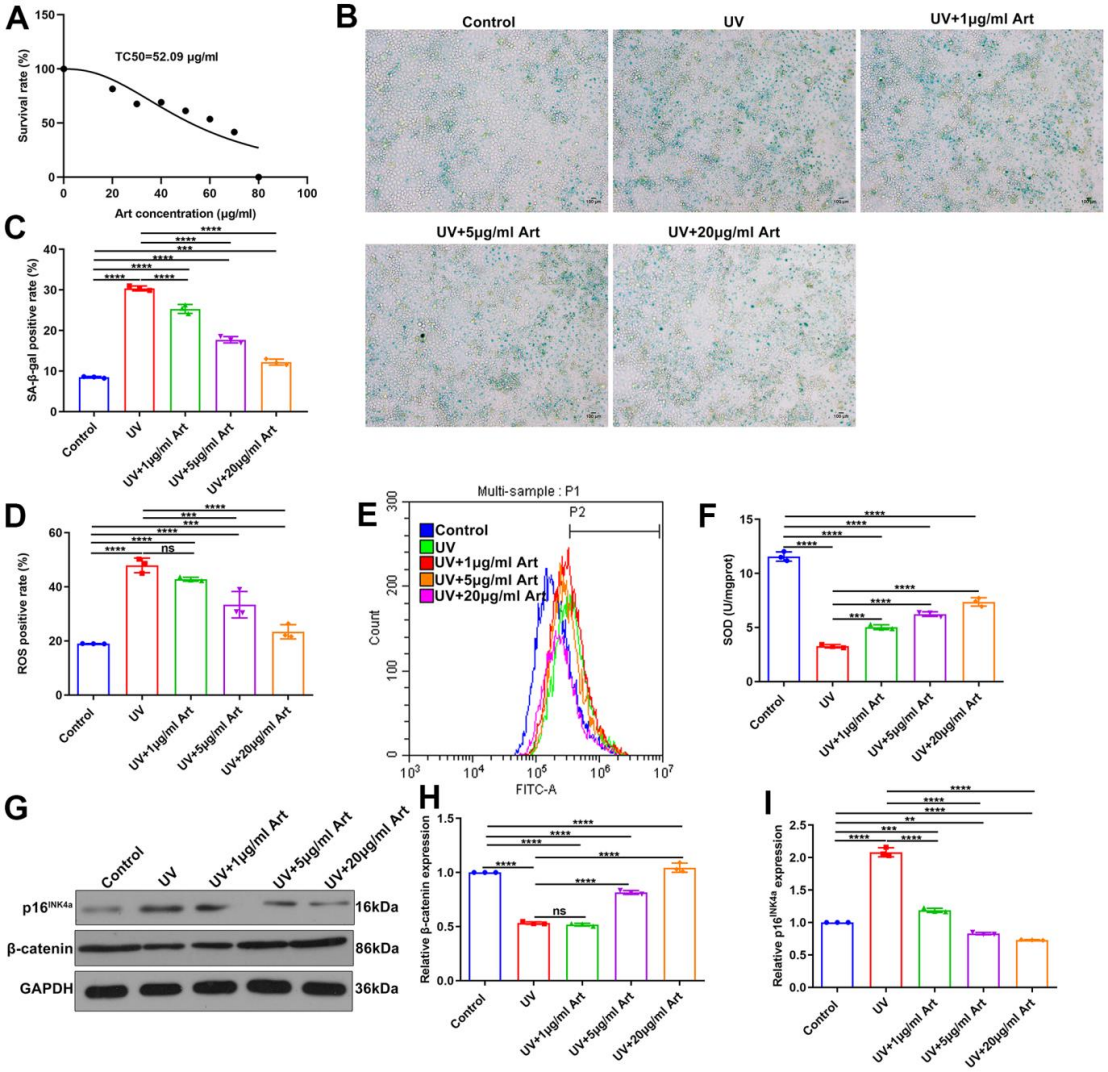
**Art pretreatment markedly reduces UVB-irradiated cell senescence, intracellular ROS production and p16^INK4a^ expression and increases SOD and β-catenin expression in HaCaT cells.** (**A**) MTT assay for determining TC50 of Art in HaCaT cells. (**B**, **C**) SA-β-gal staining for cell senescence in control group, UV group, UV + 1 μg/ml Art group, UV + 5 μg/ml Art group and UV + 20 μg/ml Art group. Scale bar: 100 μm. Magnification: 200×. (**D**, **E**) Flow cytometry of intracellular ROS production in each group. (**F**) Detection of SOD levels in HaCaT cells of different groups. (**G**–**I**) Western blot of the relative expression of β-catenin and p16^INK4a^ in each group. Ns: not significant; **p<0.01; ***p<0.001; ****p<0.0001.

To observe the therapeutic effects of Art on UV radiation-induced skin photoaging, we firstly determined the TC50 of Art in HaCaT cells. According to the MTT assay results, the TC50 value of Art was 52.09 μg/ml in HaCaT cells (Figure 1A). Based on the TC50, HaCaT cells were pretreated with three concentrations of Art (1, 5 and 20 μg/ml) for 2 h and irradiated by UVB (20 mJ/cm^2^). Control cells were cultured in the same condition without Art pre-treatment and UV radiation. After 5 days, SA-β-gal staining was utilized for detecting cell senescence. We found that SA-β-gal-positive rate was distinctly increased in UV-irradiated HaCaT cells compared to control cells (Figure 1B, 1C). But Art pretreatment significantly reduced SA-β-gal-positive rate of UV-irradiated HaCaT cells with a dose-dependent manner. Thus, Art pretreatment could effectively prevent UVB radiation-induced cell senescence. To clarify the mechanisms by which Art protected HaCaT cells against UVB radiation-induced skin photoaging, intracellular ROS levels were tested by flow cytometry. Our data showed the increased levels of intracellular ROS in UVB-irradiated cells than controls (Figure 1D, 1E). 5 and 20 μg/ml Art pretreatment distinctly decreased intracellular ROS levels in UV-irradiated cells. Nevertheless, its levels were significantly altered by 1 μg/ml Art. The mechanisms underlying Art-induced decrease of ROS were explored by measuring the major antioxidant SOD using its detection kit. In Figure 1F, SOD expression was markedly reduced after UV radiation. But Art pretreatment significantly elevated its expression in UVB-irradiated cells, with a dose-dependent manner. Collectively, Art pretreatment (especially 20 μg/ml Art) could reduce UV-irradiated cell senescence and intracellular ROS production and enhance SOD expression in HaCaT cells.

### Art treatment weakens p16^INK4a^ expression and increases β-catenin expression in UV-irradiated HaCaT cells

Western blot was performed to detect the expression of cell senescence marker p16^INK4a^ and cell growth marker β-catenin [30]. We found that p16^INK4a^ expression was significantly up-regulated in HaCaT cells after UVB radiation compared with control cells. But Art pretreatment distinctly decreased the expression of p16^INK4a^ in UV-irradiated cells, with a dose-dependent manner (Figure 1G, 1H). Lower expression of β-catenin was found in UVB-irradiated cells than controls (Figure 1G, 1I). However, its expression was significantly elevated by Art pretreatment, with a dose-dependent manner.

### Art treatment enhances cell viability and suppresses apoptosis in UV-irradiated HaCaT cells

**Figure 2:**
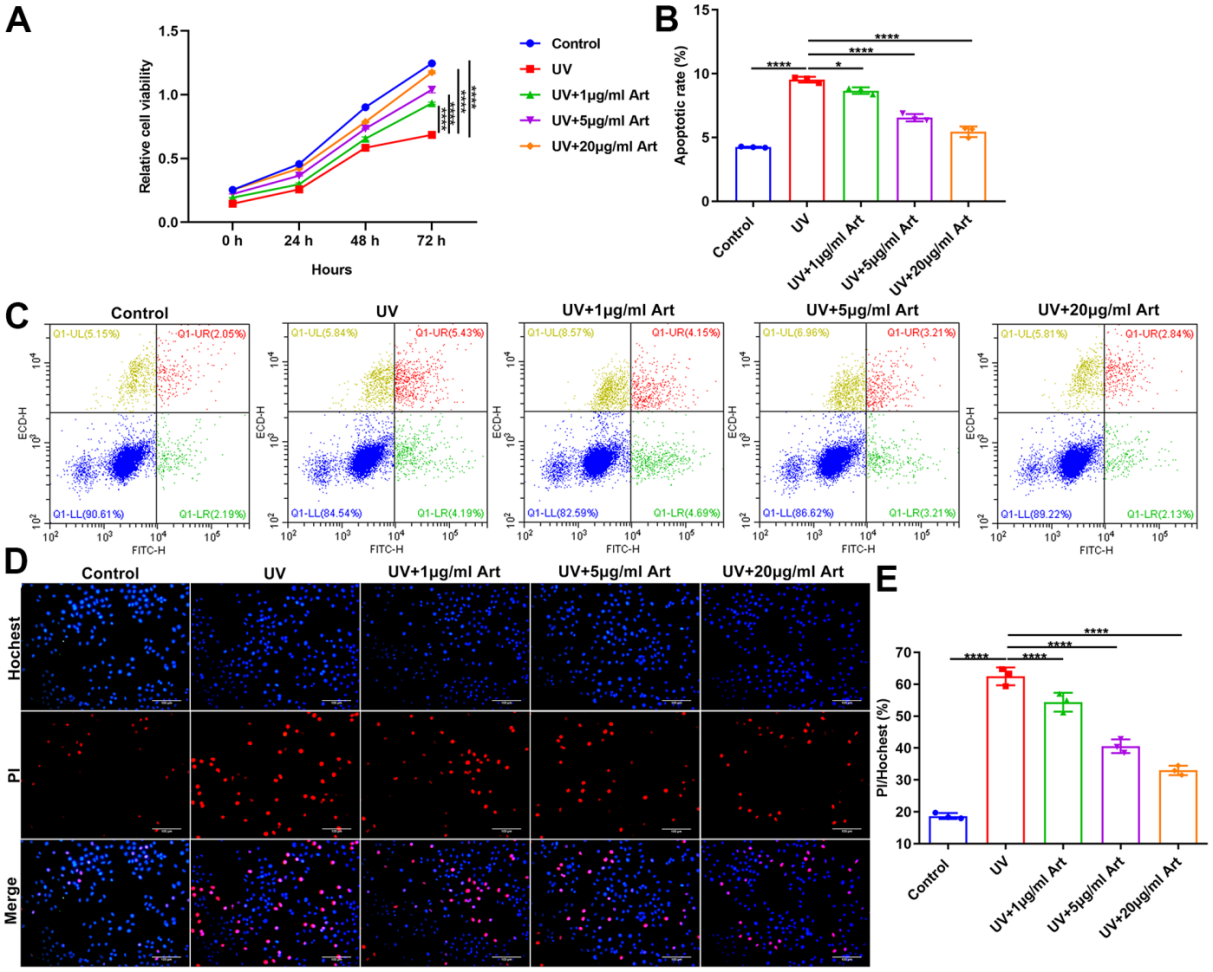
**Art treatment promotes cell viability and inhibits apoptosis in UVB-irradiated HaCaT cells.** (**A**) CCK-8 of the growth rate of HaCaT cells in control group, UV group, UV + 1 μg/ml Art group, UV + 5 μg/ml Art group and UV + 20 μg/ml Art group. (**B**, **C**) Flow cytometry of apoptotic levels in each group. (**D**, **E**) Hoechst/PI staining for morphological changes of HaCaT cell apoptosis in each group. Scale bar: 100 μm. Magnification: 200×. *p<0.05; ****p<0.0001.

At 24, 48 and 72 h after radiation, cell viability of HaCaT cells that were pretreated with 1, 5 and 20 μg/ml Art for 2 h and irradiated by UVB (100 mJ/cm^2^) for 5 days (15 min each day) was detected via CCK-8. Compared with control cells, the growth rate of UVB-irradiated cells was significantly slowed down (Figure 2A). As the concentration of Art increased, the growth rate of UVB-irradiated cells was gradually increased. This indicated that Art pretreatment may ameliorate the inhibitory effects of UVB radiation on cell growth. At 24 h of UVB radiation, cell apoptosis of each group was detected via Annexin V/PI-FITC. As shown in Figure 2B, 2C, higher apoptotic levels were found in UVB radiation group compared with control group. Art pretreatment significantly decreased apoptotic levels of UVB-irradiated cells, with a dose-dependent manner. Above findings were confirmed by Hoechst/PI staining. Under a laser scanning confocal microscope, the morphological changes of HaCaT cell apoptosis were observed. The results showed that the ratio of damaged cells was significantly increased after UVB irradiation than controls. But Art pretreatment inhibited the cell damage caused by UVB radiation with a concentration-independent behavior (Figure 2D, 2E).

### Art treatment ameliorates UV irradiation-mediated cell senescence and has no toxic side effects on normal cells

**Figure 3:**
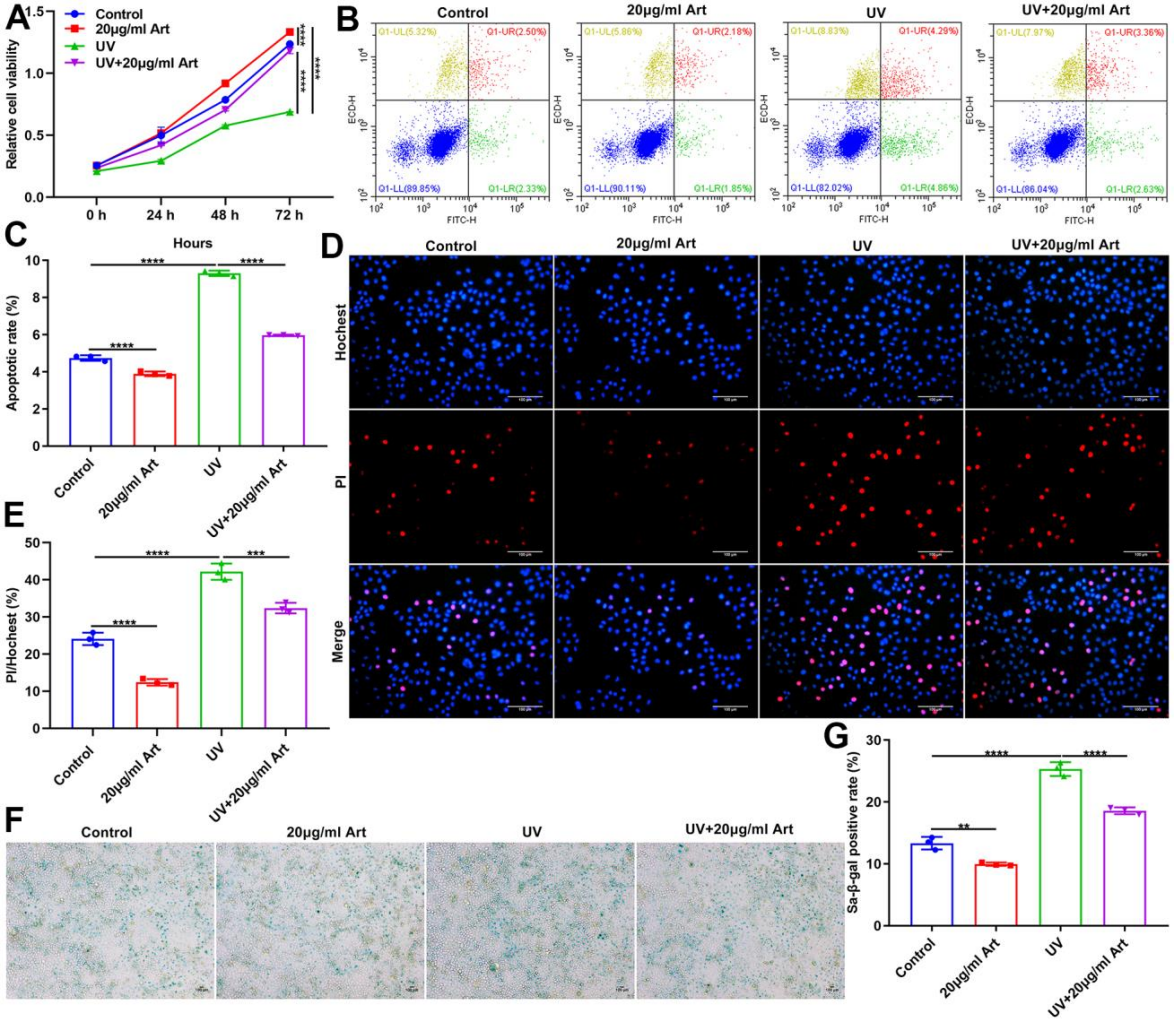
**Art treatment ameliorates UVB irradiation-induced cell senescence and has no toxic side effects on normal HaCaT cells.** (**A**) CCK-8 of the growth rate of HaCaT cells in control group, 20 μg/ml Art group, UV group, UV + 20 μg/ml Art group. (**B**, **C**) Flow cytometry of apoptotic levels in each group. (**D**, **E**) Hoechst/PI staining for morphological changes of HaCaT cell apoptosis in each group. Scale bar: 100 μm. Magnification: 200×. (**F**, **G**) SA-β-gal staining for cell senescence in each group. Scale bar: 100 μm. Magnification: 200×. **p<0.01; ***p<0.001; ****p<0.0001.

To determine Art pretreatment only had a therapeutic effect on UVB irradiation-mediated senescent cells but had no toxic side effects on normal cells, we divided HaCaT cells into control, 20 μg/ml Art, UVB radiation and UVB + 20 μg/ml Art groups. After pretreatment of 20 μg/ml Art for 2 h, HaCaT cells were irradiated by UVB for 5 days. Control cells had the same culture conditions but were not irradiated by UVB. Our CCK-8 results showed that 20 μg/ml Art pretreatment distinctly enhanced cell proliferation of not only senescent cells but also normal cells (Figure 3A). Flow cytometry was applied for detecting cell apoptosis. Our results showed that cell apoptosis was markedly increased after irradiation, but 20 μg/ml Art pretreatment significantly inhibited cell apoptosis (Figure 3B, 3C). Moreover, we found that 20 μg/ml Art pretreatment also decreased the apoptosis of normal cells. In Figure 3D, 3E, the ratio of damaged cells was significantly reduced by 20 μg/ml Art pretreatment both for UVB-irradiated cells and normal cells, indicating that Art had the effect of resisting cell damage and death. The SA-β-gal staining results showed that 20 μg/ml Art pretreatment significantly inhibited cell senescence of UVB-irradiated cells and normal cells (Figure 3F, 3G).

### Art treatment reduces cell senescence, p16^INK4a^ expression and intracellular ROS production and increases SOD expression in UV-irradiated HaCaT cells by increasing β-catenin expression

**Figure 4:**
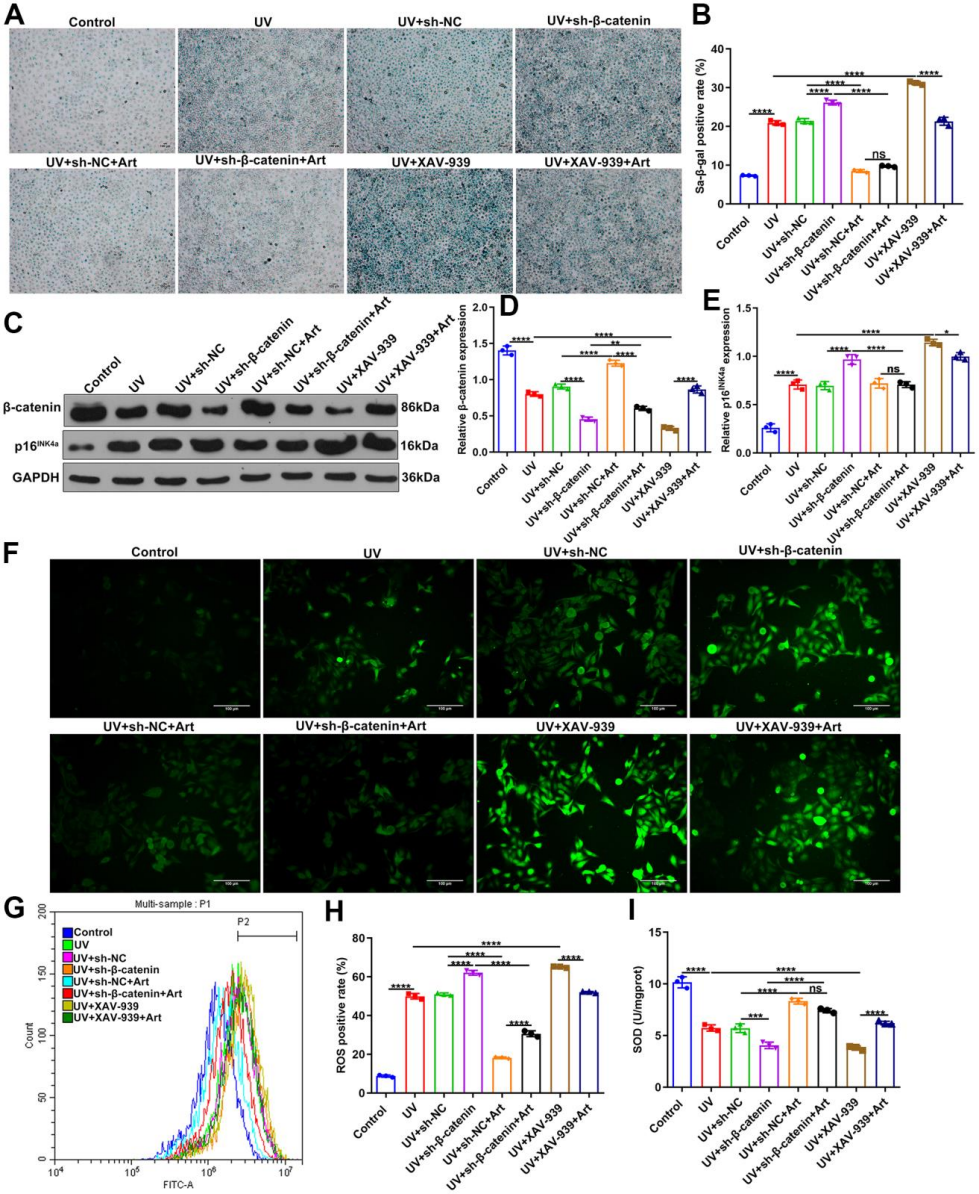
**Art treatment inhibits cell senescence, p^16INK4a^ expression and intracellular ROS production and promotes SOD expression in UVB-irradiated HaCaT cells via increasing β-catenin expression.** (**A**, **B**) SA-β-gal staining for cell senescence in control group, UV group, UV + sh-NC group, UV + sh-β-catenin group, UV + sh-NC + Art group, UV + sh-β-catenin + Art group, UV + XAV-939 group and UV + XAV-939 + Art group. Scale bar: 100 μm. Magnification: 200×. (**C**–**E**) Western blot of the expression of β-catenin and p16^INK4a^ in HaCaT cells from each group. (**F**) Immunofluorescence and (**G**, **H**) Flow cytometry of intracellular ROS expression in HaCaT cells from each group. Scale bar: 100 μm. Magnification: 200×. (**I**) Detection of SOD levels in HaCaT cells from each group. Ns: not significant; *p<0.05; **p<0.01; ***p<0.001; ****p<0.0001.

To investigate the roles of β-catenin on irradiation-driven skin photoaging, we designed shRNAs against β-catenin to silence β-catenin. XAV-939 selectively inhibits Wnt/β-catenin-mediated transcription by inhibiting tankyrase1/2, but has no effect on CRE, NF-κB and TGF-β [31]. Here, XAV-939 was selected as a positive control of sh-β-catenin. After transfection with sh-β-catenin or treatment with 10 nM XAV-939 for 24 h, HaCaT cells were pretreated with 20 μg/ml Art for 2 h and irradiated by UVB (100 mJ/cm^2^) for 15 min each day. After 5 days, SA-β-gal staining showed that sh-β-catenin significantly aggravated UVB irradiation-mediated cell senescence compared to sh-NC (Figure 4A, 4B). The similar results were observed when cells were treated with XAV-939. But sh-β-catenin did not completely affect the therapeutic effects of Art treatment on UVB irradiation-induced cell senescence. Art treatment significantly ameliorated UVB irradiation-mediated cell senescence under β-catenin silencing that was induced by sh-β-catenin or XAV-939. Western blot was presented to test the expression of β- catenin in HaCaT cells. We found that both sh-β-catenin and XAV-939 significantly aggravated the decrease in β-catenin expression in UVB-irradiated HaCaT cells (Figure 4C, 4D). Nevertheless, Art markedly elevated the expression of β-catenin under sh-β-catenin transfection or XAV-939 treatment. The expression of p16^INK4a^ was detected by western blot. As a result, both sh-β-catenin and XAV-939 significantly enhanced the expression of p16^INK4a^ in UVB-irradiated HaCaT cells, which was markedly weakened by Art treatment (Figure 4C, 4E). Immunofluorescence was applied to investigate the expression of ROS. In Figure 4F, our data showed that the increase in ROS expression after UVB irradiation, which was aggravated under transfection with sh-β-catenin or treatment with XAV-939. However, Art treatment significantly decreased ROS expression in UVB-irradiated cells that were transfected with sh-β-catenin or treated with XAV-939. Flow Cytometry was performed to verify the expression of ROS in HaCaT cells. Our data confirmed that sh-β-catenin or XAV-939 aggravated UVB irradiation-mediated increase in ROS expression, which was suppressed by Art treatment (Figure 4G, 4H). As shown in Figure 4I, UVB irradiation induced the decrease of SOD expression in HaCaT cells. Silencing β-catenin further reduced SOD expression in UVB-irradiated cells through sh-β-catenin or XAV-939, which was ameliorated by Art treatment. Collectively, Art pretreatment may suppress cell senescence, p16^INK4a^ expression and intracellular ROS production and increase SOD expression in UVB-irradiated HaCaT cells, which was closely related to the increase in β-catenin expression.

### Art treatment suppresses cell apoptosis and promotes proliferation of UV-irradiated HaCaT cells by increasing β-catenin expression

**Figure 5:**
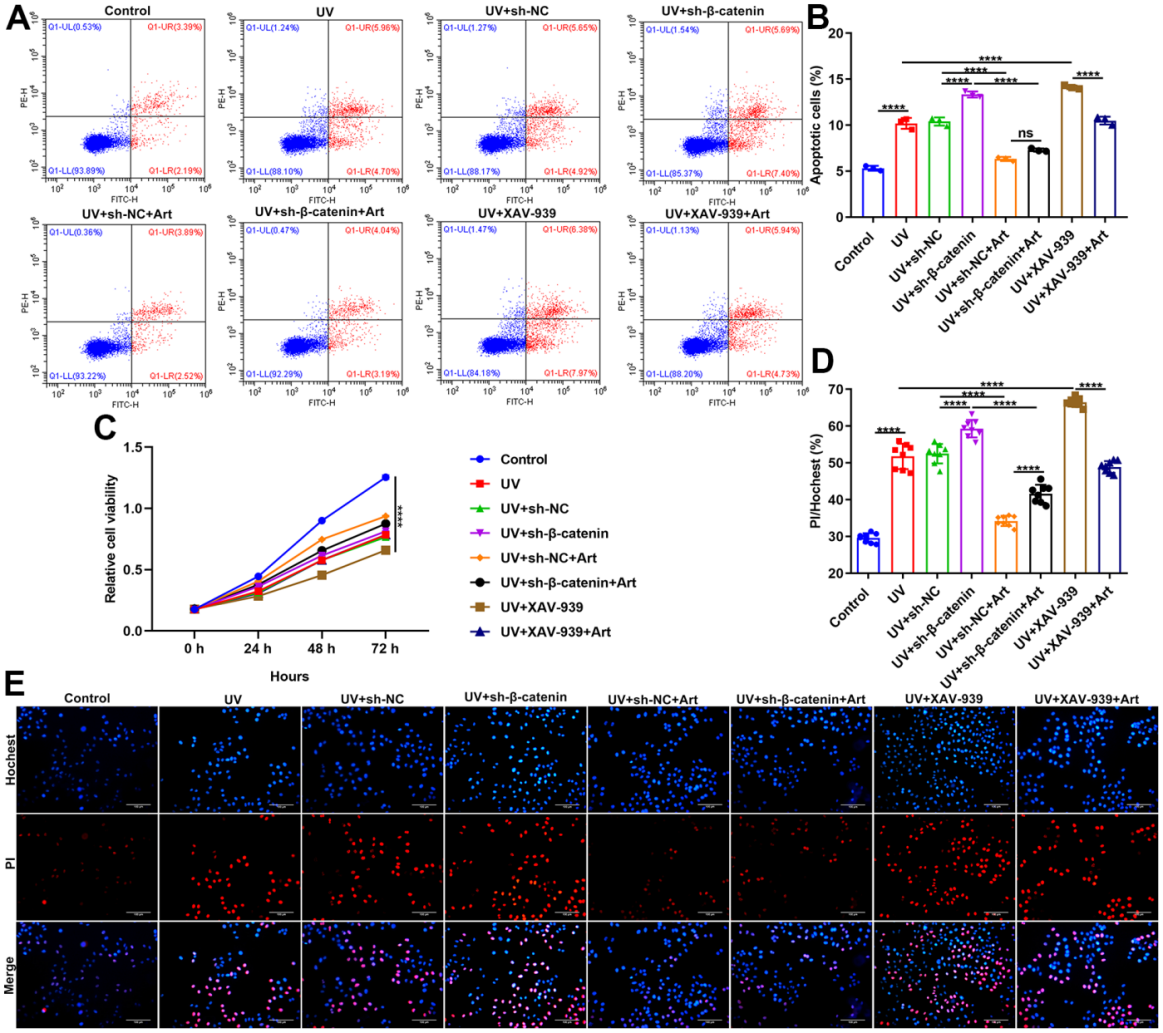
**Art treatment inhibits cell apoptosis and enhances proliferation of UVB-irradiated HaCaT cells through increasing β-catenin expression.** (**A**, **B**) Flow cytometry of apoptotic HaCaT cells in control group, UV group, UV + sh-NC group, UV + sh-β-catenin group, UV + sh-NC + Art group, UV + sh-β-catenin + Art group, UV + XAV-939 group and UV + XAV-939 + Art group. (**C**) CCK-8 assay for HaCaT cell viability in each group. (**D**, **E**) Hoechst/PI staining for morphological changes of HaCaT cell apoptosis in each group. Scale bar: 100 μm. Magnification: 200×. Ns: not significant; ****p<0.0001.

Our flow cytometry results showed that silencing β-catenin induced by sh-β-catenin or XAV-939 markedly exacerbated the apoptosis of UVB-irradiated HaCaT cells. The effects were distinctly ameliorated by Art treatment (Figure 5A, 5B). As shown in Figure 5C, UVB irradiation significantly slowed down the growth rate of HaCaT cells. The inhibitory effects were enhanced when transfection with sh-β-catenin or treatment with XAV-939. Nevertheless, Art treatment distinctly boosted the proliferation of UVB-irradiated cells with β-catenin knockdown. Hoechst/PI staining was applied for investigating the morphological changes of HaCaT cell apoptosis. Our data confirmed that UVB irradiation induced cell apoptosis (Figure 5D, 5E). Silencing β-catenin with sh-β-catenin or inhibiting β-catenin signaling pathway with XAV-939 aggravated the apoptotic effects. Art markedly alleviated the apoptosis of UVB-irradiated cells with silencing or inhibiting β-catenin pathway. Collectively, Art treatment exerted an inhibitory role on apoptosis of UVB-irradiated cells by enhancing β-catenin expression.

### Art treatment ameliorates UV-irradiated skin photoaging of mice by increasing β-catenin expression

**Figure 6:**
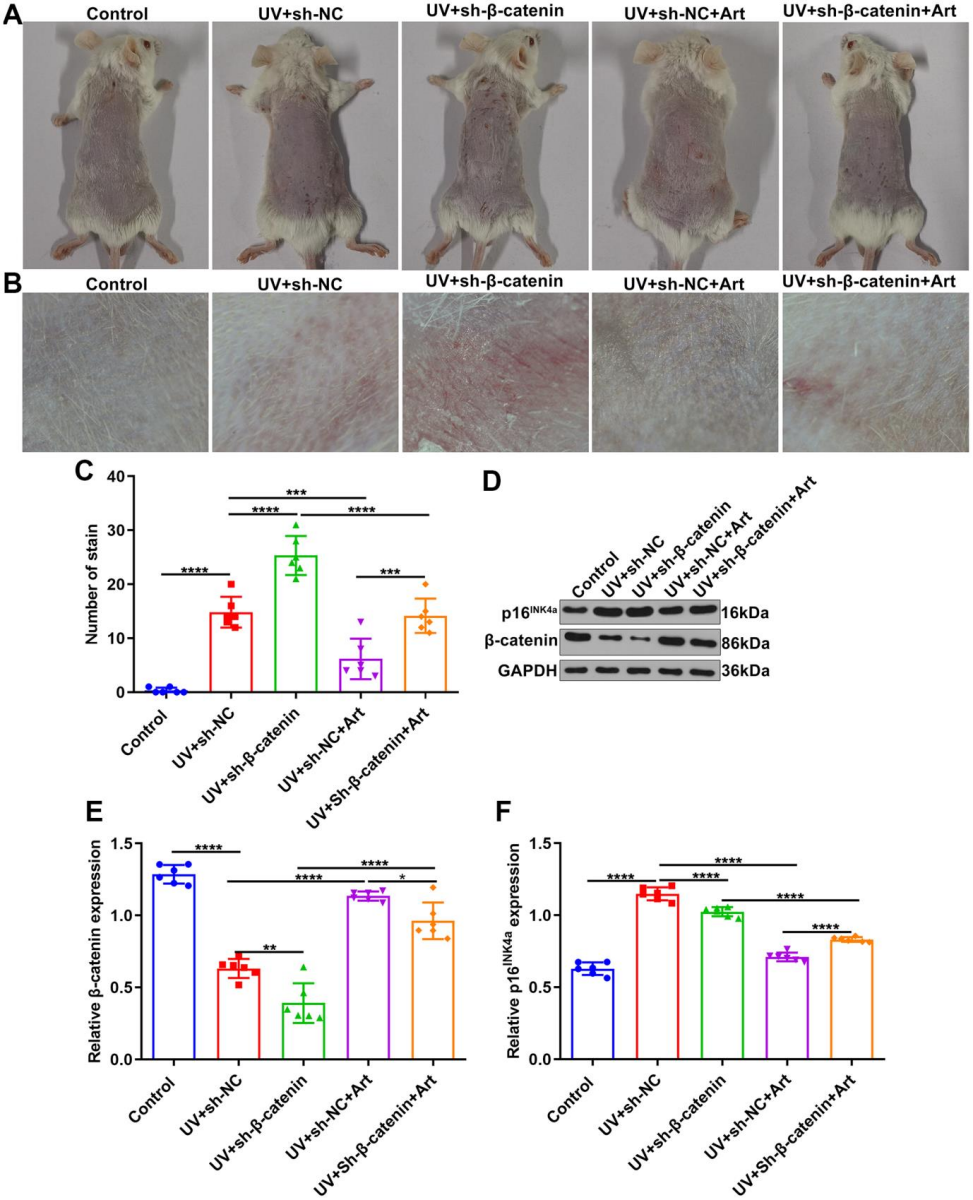
**The effects of Art treatment and silencing β-catenin expression on UVB-irradiated skin photoaging of mice.** (**A**, **B**) Clinical and dermoscopic observations of the back skin of mice in control group, UV + sh-NC group, UV + sh-β-catenin group, UV + sh-NC + Art group and UV + sh-β-catenin + Art group. (**C**) Quantification of the number of stains in skin tissue of mice from each group. (**D**–**F**) Western blot of the expression of β-catenin and p16^INK4a^ in skin tissues of mice. *p<0.05; **p<0.01; ***p<0.001; ****p<0.0001.

We further observed the effects of Art treatment and β-catenin silencing on UV-irradiated skin photoaging in mice. All mice were randomly divided into control group, UV + sh-NC group, UV + sh-β-catenin group, UV + sh-NC + Art group and UV + sh-β-catenin + Art group (n=6 each group). After 8 weeks, we observed the skin changes on the back of each group of mice. As shown in Figure 6A, 6B, the skin of the mice in the control group was smooth, normal in color, elastic, without wrinkles or sagging. The skin of the mice that were irradiated by UVB had brown patches, rough skin, wrinkle, scales, and lack of elasticity. For mice with sh-β-catenin injection and UVB irradiation, skin shrank, wrinkles deepened, brown patches and scales were prominent, and leather-like appearance appeared. After treatment with Art, skin photoaging of mice was markedly ameliorated. We also quantified the stain number in each group. As shown in Figure 6C, UV irradiation significantly increased the stain number in mice, which was enhanced by β-catenin knockdown. However, Art treatment relieved the stain number in UV-irradiated mice but the therapeutic effect of Art was weakened when β-catenin expression was suppressed. Western blot was utilized for detecting the expression of β-catenin in skin tissues of each group. After UVB irradiation, the decrease in β-catenin expression was found in skin tissues compared to controls (Figure 6D, 6E). Silencing β-catenin markedly aggravated the decrease in β-catenin expression induced by UVB irradiation. Art treatment distinctly elevated the expression of β-catenin in skin tissues of UVB-irradiated mice that were injected with sh-β-catenin. Senescence marker p16^INK4a^ was also detected via western blot (Figure 6D, 6F). The expression of p16^INK4a^ in mouse skin tissues was significantly increased after UVB irradiation. But Art treatment suppressed the expression of p16^INK4a^ in skin tissues.

### Art treatment suppresses apoptosis and intracellular ROS production and promotes SOD production in UV-irradiated skin tissues of mice by increasing β-catenin expression

**Figure 7:**
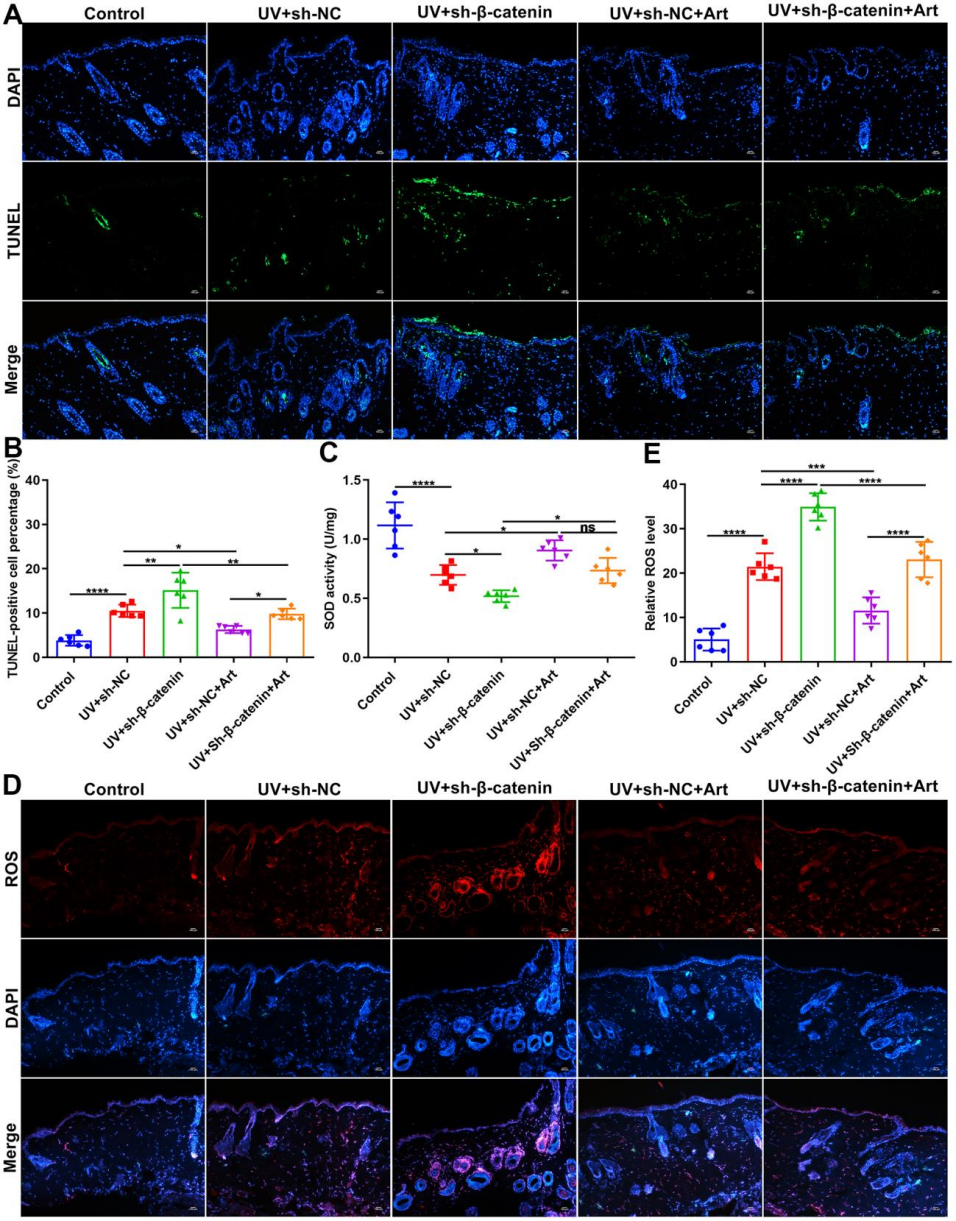
**The effects of Art treatment and silencing β-catenin expression on apoptosis and ROS and SOD production in skin tissues of mice.** (**A**, **B**) TUNEL staining of skin tissues for assessing cell apoptosis in control group, UV + sh-NC group, UV + sh-β-catenin group, UV + sh-NC + Art group and UV + sh-β-catenin + Art group. Scale bar: 100 μm. Magnification: 200×. (**C**) Detection of SOD levels in skin tissues of mice from each group. (**D**, **E**) Immunofluorescence for intracellular ROS production in skin tissues of mice from each group. Scale bar: 100 μm. Magnification: 200×. Ns: not significant; *p<0.05; **p<0.01; ***p<0.001; ****p<0.0001.

Cell apoptosis in skin tissues of mice was tested by TUNEL staining. Compared to control mice, apoptotic levels were distinctly elevated in skin tissues after UVB irradiation (Figure 7A, 7B). Silencing β-catenin significantly aggravated the apoptosis in UVB irradiated skin tissues. However, Art treatment significantly ameliorated the apoptosis of skin tissues induced by UVB irradiation and sh-β-catenin. The UVB-irradiated skin tissues were collected and ROS and SOD expression was separately detected using immunofluorescence and SOD detection kit. Compared to the matched skin area, intracellular ROS levels were distinctly elevated and SOD levels were significantly decreased in the UVB-irradiated skin tissues (Figure 7C–7E). The increase in ROS and the decrease in SOD in the UVB-irradiated skin tissues were exacerbated by silencing β-catenin expression. Nevertheless, Art treatment markedly suppressed ROS production and promoted SOD production induced by UVB irradiation and β-catenin knockdown. These data suggested that Art treatment inhibited apoptosis and ROS production as well as promoted SOD production in UVB-irradiated skin tissues of mice through enhancing β-catenin expression.

### Art treatment ameliorates UV irradiation-induced collagen fiber and elastic fiber damage in skin tissues by increasing β-catenin expression

**Figure 8:**
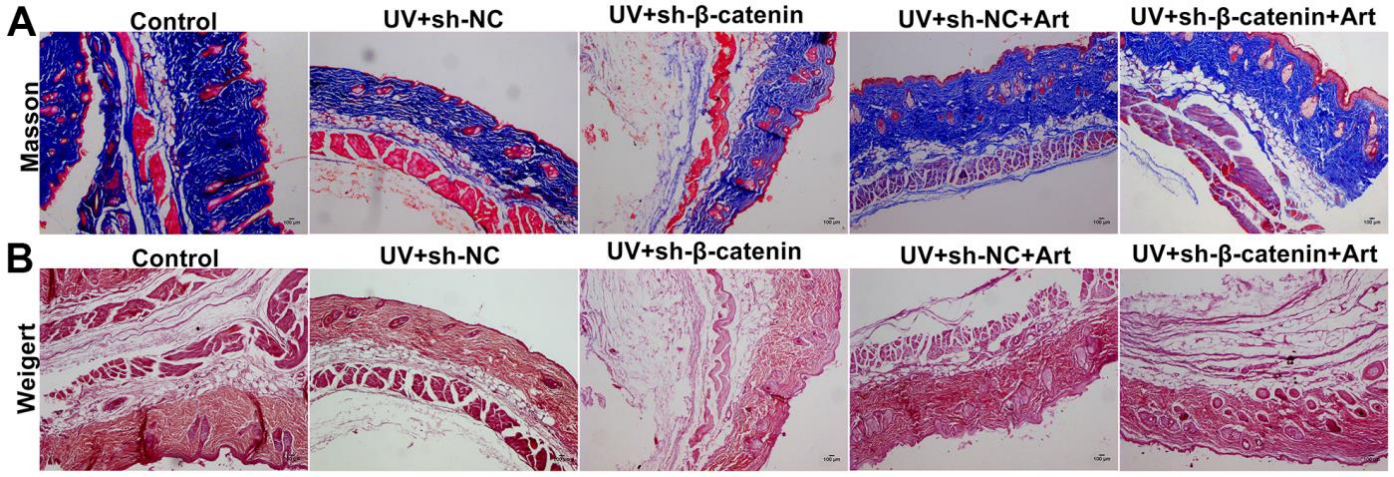
**The effects of Art treatment and silencing β-catenin expression on UV irradiation-induced collagen fiber and elastic fiber damage in skin tissues.** (**A**) Masson staining of skin tissues in each group. Control group: collagen was rich, neatly arranged without thickening or breakage; UV + sh-NC group: collagen was less and the arrangement was disordered; UV + sh-β-catenin group: there was a significant decrease in collagen and disordered arrangement; UV + sh-NC + Art group: collagen was increased, and the arrangement gradually became regular; UV + sh-β-catenin + Art group: collagen was increased slightly, but it was still thick, broken, and curled. Magnification: 100×. (**B**) Weigert staining of skin tissues in each group. Control group: elastic fibers were neat; UV + sh-NC group: some elastic fibers were broken, abnormally proliferating, crimping, bifurcation, and interweaving; UV + sh-β-catenin group: elastic fibers were significantly damaged; UV + sh-NC + Art group: elastic fiber arrangement gradually became regular; UV + sh-β-catenin + Art group: elastic fiber arrangement was slightly ameliorated. Scale bar: 100 μm. Magnification: 100×.

Masson staining was utilized for detecting collagen fiber in skin tissues of each group. In the control group, collagen fiber was arranged intact. However, we found that collagen fiber was significantly decreased in UVB-irradiated skin tissues compared to controls (Figure 8A). Furthermore, silencing β-catenin induced by sh-β-catenin significantly aggravated the decrease in collagen fiber of UVB-irradiated skin tissues. Art treatment significantly ameliorated collagen fiber degradation induced by UVB irradiation and β-catenin silencing. By Weigert staining, we evaluated elastic fiber of skin tissues. The elastic fibers on the skin in the control group were neat (Figure 8B). After UVB irradiation, part of the elastic fiber was broken, abnormally proliferating, crimping, bifurcation, and interweaving, which was aggravated by β-catenin knockdown. Nevertheless, Art treatment significantly ameliorated elastic fiber damage caused by UVB irradiation and β-catenin silencing.

## DISCUSSION

Herein, we determined the TC50 of Art as 52.09 μg/ml in HaCaT cells. According to the TC50 value, we set three concentrations of Art (1 μg/ml, 5 μg/ml, and 20 μg/ml) and investigated their effects on UV-irradiated HaCaT cells. We found that Art suppressed cell senescence, intracellular ROS production and increased SOD expression in UV-irradiated HaCaT cells with a dose-independent manner. Oxidative stress exerts a key role in both natural aging and photoaging, and this effect is strengthened during the photoaging process [32–34]. The activated photosensitizer in skin cells converts electromagnetic energy into chemical energy and combines with free O_2_ molecules to generate ROS [35]. Under normal physiological conditions, a series of antioxidant enzymes work together to maintain a low level of ROS in the body. SOD converts singlet oxygen into the less reactive compound H_2_O_2_ [36]. H_2_O_2_ has no net charge and easily reacts with Fe^2+^ through the nucleus to generate toxic hydroxyl radicals. UV-induced ROS can cause extracellular matrix damage through a series of conduction and transcription mechanisms, forming rough wrinkles, pigmentation and telangiectasia and other skin photoaging symptoms [37]. Our data suggested the roles of Art on oxidative stress induced by UV irradiation. Consistent with previous experiments, UV irradiation distinctly enhanced p16^INK4a^ expression in HaCaT cells. Art treatment reduced its expression with a dose-independent manner [38–40].

Among three concentrations, 20 μg/ml Art displayed the best inhibitory effects on UV irradiation-mediated cell senescence. Our further experiments showed that 20 μg/ml Art did not affect cell viability, apoptosis, and cell senescence of normal HaCaT cells, suggesting that 20 μg/ml Art had no toxic effect on normal cells. We also found that β-catenin expression was distinctly lowered in UV-irradiated cells, as a previous study [41]. It has been found that β-catenin signaling activation could induce melanogenesis in melanocytes [5]. Up-regulating β-catenin expression could induce migration of HaCaT cells exposed to hydrogen peroxide, thereby promoting cutaneous wound healing [42]. Our study found that Art treatment elevated β-catenin expression with a dose-independent manner, indicating that β-catenin might be a target of Art. Silencing β-catenin by sh-β-catenin or XAV-939 aggravated cell senescence, intracellular ROS production, apoptosis and p16^INK4a^ expression as well as weakened the increase in SOD expression and cell viability in HaCaT cells irradiated by UV, which was ameliorated by Art treatment. To confirm the therapeutic effects of Art on skin irradiation, we established a mouse model. Art treatment may suppress apoptosis, ROS production and collagen fiber and elastic fiber damage in UV-irradiated skin tissues of mice by increasing β-catenin expression. However, a limitation of animal experiments should be pointed out. Herein, to study the effect of β-catenin on photoaging *in vivo*, sh-β-catenin adenovirus was injected between the dermis and epidermis of the irradiated site once a week for the mice. However, the specificity of this method is limited as all the cells in this area would be interfered with by the virus, not just the epithelial cells used by the authors in the *in vitro* experiment. In our future, Cre/Floxed knockout mice will be used to verify our conclusion.

Collectively, this study suggested that Art treatment exerted an inhibitory effect on skin photoaging both in UV-irradiated HaCaT cells and mouse models. Further analysis found that β-catenin was responsible for UV irradiation-mediated skin photoaging. Mechanically, Art treatment ameliorated skin photoaging by enhancing β-catenin expression. Therefore, our findings offered novel clues for pharmacological activity of Art.

## MATERIALS AND METHODS

### Cell culture

Human immortalized epidermal cells HaCaT were purchased from iCell company (Shanghai, China). The cells were cultured in DMEM medium that contained 10% fetal bovine serum (GIBICO, USA) and 1% penicillin-streptomycin double antibody. They were maintained in a humidified incubator with 5% CO_2_ at 37° C.

### Methyl thiazolyl tetrazolium (MTT) assay

The median toxic concentration (TC50) of Art was detected in HaCaT cells by MTT assay. HaCaT cells were spread on a 96-well plate, and when the cell density was 70%, 8 concentration gradients (0, 0.0025, 0.005, 0.01, 0.02, 0.04, 0.08, 0.16 μg/μl) of Art were set and separately added to cells. After 48 h, 20 μL 5 g/L MTT was added to each well and continued to culture for 4 h. Then, 150 μL DMSO was added to each well. The absorbance value was detected with a microplate reader at a wavelength of 490 nm. According to the cell viability, concentration gradients were further divided into 0, 20, 40, 60 and 80 μg/ml. The cell viability was then detected by MTT method and TC50 of Art was calculated. Based on the TC50 value, low (1 μg/ml), medium (5 μg/ml) and high (20 μg/ml) concentration of Art were finally determined.

### UV radiation and treatment

HaCaT cells were irradiated with ultraviolet B (20 mJ/cm^2^) to induce cell senescence, UBV irradiation lasted for 5 days, once a day. The cells were pretreated with different concentrations of Art (1, 5 and 20 μg/ml) for 2 h before irradiation.

### Senescence-associated β-galactosidase (SA-β-gal) staining

The HaCaT cells were detected by SA-β-gal staining kit (C0602; Beyotime, Shanghai, China). 1 ml SA-β-gal staining fixative was added to make it fully cover the surface of the sample, which was placed in a wet box for 15 min at room temperature. Then, the sample was rinsed 3 times with PBS for 5 min each time. The filter paper was used to absorb water, and an appropriate amount of staining working solution was added to make the working solution completely cover the edge of the sample. The slides were put in a wet box in a 37° C thermostat overnight. The collected images were observed under a microscope (Olympus, Japan). Senescent cells were stained dark blue.

### ROS detection

The supernatant of HaCaT cells was discarded and the cells were washed once with PBS. The, the cells were incubated with DCFH-DA fluorescent probe (CA1410; Solarbio, Beijing, China) for 30 min. After aspirating the probe staining solution, the cells were washed with PBS and trypsinized. The collected cells were centrifuged at 1100 g for 3 min. After discarding the supernatant, the cells were resuspended in PBS and centrifuged at 1100 g for 3 min. After aspirating the liquid, PBS was added to resuspend the cells. Finally, ROS levels were detected using flow cytometry. According to the instructions of ROS kit, immunofluorescence of ROS was also carried out. The expression of ROS was investigated under a fluorescence microscopy (Olympus, Japan).

### Detection of SOD levels

The SOD detection process was carried out strictly in line with the instructions of SOD detection kit (S0101S; Beyotime, China). Briefly, HaCaT cells were planted onto a 12-well plate (2 × 10^5^ cells / mL) and the cell lysates were prepared with PBS. Skin tissues were placed in cold saline and homogenized through a homogenizer machine, followed by centrifugation at 3000 g lasting 15 min to obtain the supernatant. SOD levels were detected in HaCaT cells and 10% skin tissue homogenate.

### Western blot

Western blot was used to examine the expression of β-catenin and p16^INK4a^ proteins in HaCaT cells or skin tissues. After the cells or tissues were lysed and centrifuged, the total protein was extracted. According to the measured protein molecular weight, 12% separating gel and 5% concentrated gel were configured separately. The sample was loaded at an amount of 50 μg/porin. Electrophoresis was performed, and then the sample was transferred onto the membrane. After being removed from the blocking solution, the membrane was incubated with primary antibody against β-catenin (1:1000; ab32572; Abcam, USA), p16^INK4a^ (1:1000; 10883-1-AP; Proteintech, USA) and GAPDH (1:1000; 60004-1-Ig; Proteintech, USA) overnight at 4° C. After washing the membrane, the secondary antibody (1:1000; ab7090; Abcam, USA) was incubated at room temperature for 1 h. The protein was developed by ECL kit and the grey value was quantified by ImageJ software (version 1.48).

### Cell counting kit-8 (CCK-8)

HaCaT cells (5×10^3^ cells/well) were seeded in a 96-well culture plate, with 100 μl of cell culture medium in each well. Each group had 3 replicate wells. After the cells were cultured for 24 h, 10 μl of CCK8 reagent (Dojindo, Japan) was added to each well, incubated at 37° C for 4 h. The absorbance was measured at 450 nm.

### Flow cytometry for apoptosis

Apoptosis of HaCaT cells was detected by cell apoptosis detection kit (CA1020; Solarbio, Beijing, China). HaCaT cells were digested with trypsin and adjusted to 1×10^6^/ml. 1 ml of cells was taken from each group, washed 3 times with pre-cooled PBS, and resuspended the cells in 200 μl buffer. Then, the cells were incubated with 10 μl Annexin V fluorescein isothiocyanate and 10 μl propidium iodide for 30 min at 4° C, followed by being added by 300 μl binding buffer. The apoptosis rate was detected by flow cytometry.

### Hoechst/PI staining

Hoechst/PI detection kit (CA1120; Solarbio, Beijing, China) was applied for cell apoptosis. HaCaT cells were incubated with 1 mg/ml Hoechst 33258 solution at 37° C for 7 min. After cooling on ice, the cells were centrifuged, and resuspended in PBS. Then, the cells were incubated with 5 mg/ml PI dye solution. After centrifugation, the cells were washed once with PBS. The morphological changes of HaCaT cell apoptosis were observed under the laser confocal microscope.

### TdT-mediated dUTP nick-end labeling (TUNEL) staining

Cell apoptosis was detected via TUNEL staining detection kit (11684817910; Roche, Shanghai, China). According to the kit instructions, TUNEL staining of HaCaT cells and skin tissues was carried out. The positive cells were observed under a fluorescence microscopy.

### Cell transfection

According to the sequence of β-catenin, shRNA against β-catenin (sh-β-catenin; Wuhan GeneCreate Biological Engineering Co., Ltd., Wuhan, China) was designed and packaged adenovirus (titer 1*10^11^ PFU/ml, 1ml), and at the same time packaged shRNA negative control (sh-NC; Wuhan GeneCreate Biological Engineering Co., Ltd., Wuhan, China) adenovirus. The sequences of sh-β-catenin and sh-NC were as follows: sh-β-catenin: 5’-GTTGTTATCAGAGGACTAAATATTCAAGAGATATTTAATGTCCTCTGATAACAATTTTT-3’ and sh-NC: 5’- CACCGTTCTCCGAACGTGTCACGTCGAAACGTGACACGTTCGGAGATTTTT-3’. After silencing β-catenin for 24 h, the cells were treated with 20 μg/ml Art, followed by UV irradiation. 10 nM XAV-939 (HY-15147; Medchemexpress, Beijing, China) was used as a positive control of sh-β-catenin.

### Animals

30 BALB/c mice were purchased from Charles River Company (Shanghai, China). The breeding conditions were Specific pathogen Free (SPF). The temperature of the mouse breeding environment was (23±2° C), the relative humidity was 60%, the day and night were alternated for 12/12h, and feed and water were freely available. This study strictly complied with the ethical standards set by the Animal Ethics Committee of Hubei University of Chinese Medicine (8217152303). After 1 week of adaptive feeding, these mice were randomly separated into five groups (6 each group): control; UV + sh-NC, UV + sh-β-catenin, UV + sh-NC + Art, UV + sh-β-catenin + Art. According to the clinical dosage, Art was dissolved in sterilized water and fed by mice (0.2 ml/10 g). 1*10^9^ PFU sh-β-catenin adenovirus was injected between the dermis and epidermis of the irradiated site once a week. The mice were irradiated for 8 weeks. UVB radiation at equivalent surface exposure (100 mJ/cm^2^) three times per week, the skin tissue of the irradiated area was collected.

### Masson staining

Fresh skin tissue was soaked in 4% paraformaldehyde tissue fixative. After 72 h of fixation at room temperature, the tissue was dehydrated by conventional methods and embedded in paraffin in the longitudinal direction of the skin. The slices were 4 μm thick. The collagen fibers of paraffin section were dyed blue with aniline blue, and collagen fibers was observed under a microscope.

### Weigert staining

Fresh skin tissue was fixed with 10% neutral formalin and embedded in paraffin. The slices were 4 μm thick, and routinely deparaffinized to water. The slices were stained with Weigert (G1590; Solarbio, Beijing, China) and hematoxylin for 15 min, followed by being washed back to blue for 5 min. Then, the slices were stained with VG staining solution for 1 min. After discarding the dye solution, 95% alcohol solution was used for rapid differentiation for a few seconds. Then, the slices were dehydrated with absolute ethanol, transparent xylene and sealed. The images were finally observed.

### Statistical analysis

The data were analyzed by Graphpad 7.0. All quantitative data were displayed as the means ± standard deviation. One-way analysis of variance was used for multiple comparisons. P<0.05 was considered statistically significant.
